# Growth Characteristics of a *Desmodesmus* Species from the San Antonio Springs and Its Short-Term Impact on Soil Microbial Dynamics

**DOI:** 10.3390/life14091053

**Published:** 2024-08-23

**Authors:** Lauren K. Bomer, Betsy D. Leverett

**Affiliations:** 1 Marine Science Institute, The University of Texas at Austin, 750 Channel View Drive, Port Aransas, TX 78373, USA; lauren.bomer@utexas.edu; 2 Department of Chemistry and Biochemistry, University of the Incarnate Word, 4301 Broadway, San Antonio, TX 78209, USA

**Keywords:** microalgae, soil ecological health, agriculture

## Abstract

A new *Desmodesmus* species was isolated from the largest of the San Antonio Springs, the Blue Hole, in San Antonio, Texas, and characterized for its potential applications in sustainable agriculture. The xenic isolate (*XB*) was established by enrichment and subcultured to produce the axenic isolate (*AxB*), which was identified based on morphological features and DNA profiling, confirming its close phylogenetic relationship with *Desmodesmus* spp. Growth characteristics, biomass composition, and pigment profiles were assessed for both the xenic and axenic isolates along with their growth in saline conditions and a range of seasonal Texas temperatures. Both *Desmodesmus XB* and *Desmodesmus AxB* exhibited optimal growth at 25 °C as well as robust growth at 37 °C and in weakly saline media (5 g/kg NaCl). Biomass analysis revealed levels of carbohydrates, proteins, lipids, chlorophylls, and carotenoids comparable to other desmids and pigment profiles supported the *Desmodesmus* classification. Soil studies demonstrated the persistence of *Desmodesmus XB* and influence on microbial activity, indicating the potential of this isolate for agricultural applications such as soil remediation.

## 1. Introduction

Microalgae have become an increasingly important focus in the development of sustainable agriculture and environmental remediation due to their adaptability as biofertilizers and biostimulants [1], remediators of municipal and industrial wastewater [2], and CO_2_ sequestration agents [3]. The effectiveness of microalgal biomass in decreasing agrochemical dependence and in recapturing nutrients from marginal water resources such as agricultural runoff, coupled with their ability to accumulate heavy metals and to produce renewable fuels from wastewater, have highlighted their potential importance in an emerging bioeconomy based on resource use efficiency and recycling [4,5,6].

The agronomic significance of microalgal interactions with the soil microbiome is an active area of research, and microalgae-based improvements in soil fertility and soil health have been recently reviewed [7,8]. Microalgae and cyanobacteria improve soil microbial diversity [9], promote plant growth [10], cooperate with other soil microbes to detoxify agrochemicals [11], and bioremediate saline and heavy-metal-contaminated soils [12,13]. More recently, special attention has been paid to microalgal consortia and to the sustainable soil benefits of mixed microbial communities that include microalgae [9,14,15]. Extremophile algae have also garnered particular interest in soil remediation research since microalgae that thrive in extreme environments have shown remarkable capacities for heavy metal accumulation [16], toleration of extremes in temperature and salinity [17], and production of high value products under biotic or abiotic stresses [18,19].

Microalgae are a large and diverse taxonomic group, with estimates ranging from 70,000 to over a million species [20,21], and although these organisms collectively provide nearly half of the global net primary production in marine and freshwater systems, they remain mostly unstudied outside of a few thousand species [22]. Bioprospecting in unique aquatic and terrestrial ecosystems for new microalgae remains an effective strategy to expand microalgal taxa and, when used in conjunction with an operational screening platform [17,23,24,25], can facilitate the identification of new algal species with the potential to improve agricultural and environmental resilience and sustainability.

**Figure 1:**
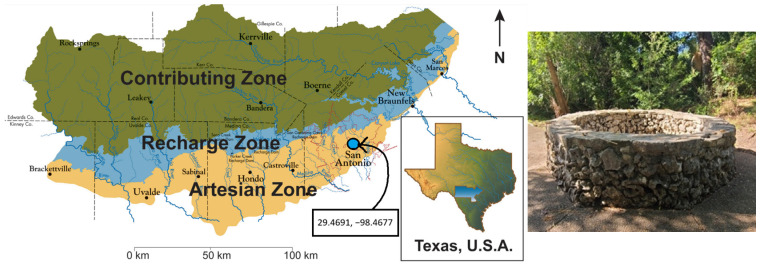
(**left**) Map of the Edwards Aquifer region showing the GPS coordinates for the *Desmodesmus* sp. isolation and the three zones of the aquifer: the Contributing Zone, the Recharge Zone, and the Artesian Zone [26]. (**right**) The largest of the San Antonio Springs, the Blue Hole, with its octagonal stone well structure.

The Edwards Aquifer is one of the largest artesian reservoirs in the world, spanning eight counties in south central Texas and serving as the primary water source for more than 2.5 million people (Figure 1). The southern border of the aquifer is formed by its artesian zone, so named for the numerous springs of varying size that have historically connected the aquifer to local rivers such as the Comal, the Guadalupe, and the San Antonio [26]. As demands on the aquifer have increased, the springs across the artesian zone have become dry more often during the year and increasingly do not flow except during extremely rainy periods of sustained recharge in the aquifer. The San Antonio Springs is a group of springs in north central San Antonio, the largest of which is known colloquially as the Blue Hole. The Blue Hole was chosen for algae prospecting because of its variable periods of desiccation and because of its distinctive color when it is flowing. The goal of the present work has been to isolate, identify, and characterize a native microalga from the dynamic environment of the San Antonio Springs and to examine its potential agricultural advantages through its growth characteristics, its responses to saline and temperature stress, and its impacts on soil microbial activity and functional diversity.

## 2. Materials and Methods

### 2.1. Chemicals, Culture Media, and Reference Algae

Chemicals and media stock solutions were purchased from Millipore Sigma (Burlington, MA, USA) unless otherwise stated. *Tisochrysis lutea* was used as a reference organism for pigment analysis and was purchased from Bigelow NCMA (East Boothbay, ME, USA). Other reference algae, *Pleurochloris mierengensis* and *Scenedesmus dimorphus*, and GR+ soilwater medium were purchased from the UTEX Culture Collection of Algae (Austin, TX, USA). BG11, MB3N, and F/2 culture media were prepared according to the recipes of the UTEX culture collection, with the exception that GR+ soilwater medium was added to all three media types at the rate prescribed for MB3N media (40 mL/L).

### 2.2. General Procedures for Algae Culturing, Harvest, and Quantitation

Algae cultures were maintained as axenically as possible through regular passage in the appropriate sterile media using a circulation-free workstation (Mystaire, Inc., Creedmoor, NC, USA). New algal isolates were maintained in BG11 medium, *T. lutea* was maintained in F/2 medium, and *S. dimorphus* and *P. mierengensis* were maintained in MB3N medium. All cultures were grown in an open, multi-tier greenhouse under cool white light (40 mmol/m^2^ s) and aerated either by orbital rotation or by sterile-filtered air bubbled into the cultures. Algae were harvested by centrifugation, and the resulting biomass pellets were weighed (fresh weight (FW) and either stored at −20 °C or dried at 42 °C for 120 h for dry weight (DW) biomass determination. Microscopic observations to monitor the general health status of cultures and manual quantitation procedures were performed using either an Olympus CKX41 (Evident Scientific, Waltham, MA, USA) inverted microscope or a National B2-220 (National Optical, Schertz, TX, USA) light microscope. Algal cell densities were determined as prescribed in previous reports [27,28,29] by duplicate manual counting using a Neubauer chamber and by correlation to (a) duplicate automatic counting using a TC-20 (Bio-Rad, Hercules, CA, USA), (b) optical density measurement at 540 nm in triplicate, or (c) chlorophyll fluorescence (ex.450/em.680) observed in triplicate using an Infinite 200 Pro plate reader (Tecan USA, Morrisville, NC, USA). Initial assessments of the exponential growth period and initial doubling times for each culture under laboratory conditions were also made using these general quantitation procedures.

### 2.3. Isolation of Xenic and Axenic Cultures

The alga used in this study was isolated from the Blue Hole (29.469076315241015, −98.46767723558234) during a flow period following heavy rains. Water samples were collected and enriched (1:1) with BG11 medium and then maintained under cool white light (40 mmol/m^2^ s), applying a 16:8 h light–dark photoperiod and aerating the culture by rotation. An enriched culture was initially designated *BlueC* (referred to as *xenic Desmodesmus* or *XB*) and established on a one-liter scale. *BlueC* culture was then iteratively grown on 1% agar made with BG11 medium, and individual colonies were selected and subcultured in fresh BG11 medium to establish an axenic liquid culture initially designated *Blue 3.1* (referred to as *axenic Desmodesmus* or *AxB*). Both *XB* and *AxB* cultures have been maintained in liquid culture and on 1% agar continuously since their establishment and harvested as described in the previous section.

### 2.4. Species Identification and Phylogenetic Analysis

The axenic isolate, *AxB*, was submitted to the UTEX Culture Collection for genus-level identification through morphological examination and phylogenetic analysis of the internal transcribed spacer (ITS) rDNA according to their established methods [30,31]. Briefly, DNA was extracted from *AxB* culture using a manual disruption protocol and the detergent, dodecyltrimethylammonium bromide (DTAB) [30], and PCR was performed using primers described by Goff [31] to amplify the ITS1 and ITS2 rDNA regions. The PCR products were sequenced and used to generate a consensus sequence from three bidirectional sequence pairs using Geneious software (Geneious Prime^®^ v2023.2.1, Build 20 July 2023 11:29). The obtained sequence was submitted to NCBI Blast analysis to determine its preliminary affiliation, and a phylogenetic tree was constructed through Maximum Likelihood and Neighbor Joining analyses using Geneious Prime 2023.2.1 with the Clustal Omega alignment tool and the Geneious Tree Builder plugin (Tamura-Nei nucleotide substitution model, (https://www.geneious.com). A *Desmodesmus* representative alga, KP645234.1 *Acutodesmus obliquus*, was selected as the root of the phylogenetic tree based on the identity of the nearest neighbor groups.

### 2.5. Growth Rate Determination

Growth rate analysis was performed in 1 L sterile glass flasks for each of the new isolates, *Desmodesmus XB* and *Desmodesmus AxB*, for a period of five days to observe exponential growth for the calculation of their growth rates and doubling times. Microscopic observations were performed using either an Olympus CKX41 (Evident Scientific, Waltham, MA, USA) inverted microscope or a National B2-220 (National Optical, Schertz, TX, USA) light microscope. Algal cell densities were determined as described in Section 2.2 by duplicate manual counting using a Neubauer chamber with correlation to duplicate automatic counting using a TC-20 (Bio-Rad, Hercules, CA, USA). Algae were harvested by centrifugation on Day 5 of the growth experiment, and both fresh weight (FW) and dry weight (DW) of algal cell biomass produced in each 1 L culture were determined as described in Section 2.2.

### 2.6. Biomass Processing and Estimation of Carbohydrates, Proteins, and Lipids

**Figure 2:**
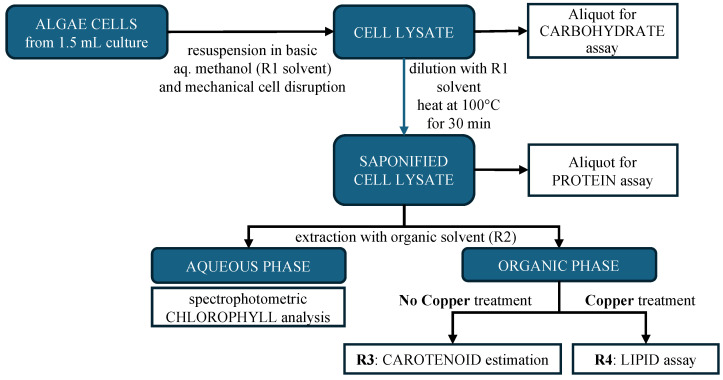
Summarized procedures for the combined compositional analysis of algal biomass [32].

Compositional analysis of *XB* and *AxB* biomass was performed on actively growing algal cells using the extraction method of Chen and Vaidyanathan [32] modified with assay procedures to estimate carbohydrate, protein, and lipid contents [33,34]. The extractive procedure and sample generation used for these studies is summarized in Figure 2. Initially, a 1.5 mL aliquot of algae culture (O.D. 0.5–1.5 at 540 nm) was centrifuged (3000 rpm, 5 min), and the pellet was redissolved in 0.02 mL phosphate buffer (50 mM, pH 7.0) and 0.48 mL of the R1 solvent (1M NaOH in 25% methanol). This basic cell suspension was subjected to bead milling for 14 min using a BeadBug 6 Homogenizer (Benchmark Scientific, Sayreville, NJ, USA), and aliquots of the lysate (0.2 mL) were set aside for carbohydrate analysis. The remaining lysate was diluted by addition of an additional 1 mL of R1 solvent, and the mixture was heated at 100 °C for a 30 min saponification. Aliquots removed after saponification were used for protein analysis. A 0.5 mL aliquot of the remaining lysate was transferred to a 2 mL tube containing 0.75 mL of the R2 solvent (chloroform/methanol, 2:1) for liquid–liquid separation of chlorophylls from lipids and carotenoids. The final organic phase from the R2 extraction was subjected to treatment with an equal volume of either TEA buffer (triethanolamine/acetic acid, 9:1) or Cu-TEA buffer (triethanolamine/acetic acid/6.25% Cu(NO_3_)_2_, 9:1:10) to produce the R3 and R4 samples, respectively, for each culture.

The R3 sample was used to estimate carotenoid content, and the R4 sample was used to estimate lipid content. Carbohydrate content was assayed according to Masuko [33] using glucose standards prepared in R1 solvent, and protein was determined using the Bradford reagent (BioRad, Hercules, CA, USA) according to the manufacturer’s protocol but with standardization to bovine serum albumin in R1 solvent. Lipids and carotenoids were estimated as described by Chen and Vaidyanathan [32] using the absorbance of the final R4 organic phase at 260 nm to observe the Cu-TEA–fatty acid complexes formed in the extraction process [34] and the carotenoid absorbance maxima at 430, 450, and 480 nm. Carotenoids were also examined qualitatively as part of the pigments analyses described in the next section.

### 2.7. Pigment Extraction and Analyses

Three methods for chlorophyll and carotenoid extraction and analysis were performed to ensure a more accurate examination of chlorophyll and pigment content. First, the alkaline saponification and organic extraction summarized in Figure 2 was used to separate chlorophylls away from carotenoids and into the aqueous phase for less ambiguous spectral analysis of both phases [32]. The organic phase samples designated R3 (Figure 1) were observed in duplicate (350–800 nm) using an Infinite 200 Pro plate reader (Tecan USA, Morrisville, NC), and carotenoid content was estimated as previously described [32]. The absorbance of the aqueous phase chlorophylls (350–800 nm) was observed in triplicate using an Infinite 200 Pro plate reader, and the relevant chlorophyll concentrations were calculated from the following reported expressions [32]:

C_a_ = 6.40 × A_416_ − 0.79 × A_453_ (1)

C_b_ = 5.87 × A_453_ − 0.24 × A_416_ (2)

where C_a_ is chlorophyll a, and C_b_ is chlorophyll b. A second extraction method [35] was utilized to provide more conventional absorption spectra in 80% acetone for verification of the types of chlorophyll present in *XB* and *AxB*. Briefly, a 2 mL aliquot of culture was harvested, washed with distilled water, and centrifuged (5000 rpm, 5 min). The pellet was resuspended in 0.4 mL of 50 mM phosphate buffer (pH 7) and subjected to bead milling for 14 min using a BeadBug 6 Homogenizer (Benchmark Scientific, USA). Following the addition of 1.6 mL of acetone, the lysate was vortexed vigorously for 2 min, incubated in the dark for 15 min, and centrifuged (5000 rpm, 5 min). The absorbance of the resulting supernatants (350–800 nm) was observed in triplicate using an Infinite 200 Pro plate reader (Tecan USA, Morrisville, NC, USA), and the chlorophyll content was calculated using the Formulas (3)–(5) below [35]:

C_a_ = 12.21A_663_ − 2.81A_646_ (3)

C_b_ = 20.13A_646_ − 5.03A_663_ (4)

(5) $$C_{t}=\frac{1000A_{470}-3.27C_{a}-104C_{b}}{198}$$

where C_a_ is chlorophyll a, C_b_ is chlorophyll b, and C_t_ is the total carotenoids (g/mL). The third extraction method was a modification of the second method involving the extraction of more culture (10 mL) to provide a more concentrated extract of chlorophylls and carotenoids for separation using thin-layer chromatography (TLC). In this method, 10 mL of algae culture (O.D. at 540 nm = 0.5–1.5) was extracted according to Wellburn [35], then 0.010 mL of each of the resulting extracts was subjected to TLC using normal phase TLC plates with a hexane/acetone (7:3) running solvent in accordance with previous reported methods [36,37,38]. Several reference algae, *T. lutea*, *S. dimorphus*, and *P. mierengensis*, were also extracted using this third method to provide chromatogram comparisons with known chlorophyll and carotenoid profiles [39,40,41].

### 2.8. Salt and Temperature Tolerance Experiments

The tolerance of *XB* and *AxB* cultures to growth in hypersaline conditions (5, 10, 20, and 30 g/kg NaCl in BG11 medium) was examined across a range of seasonal Texas temperatures (15, 25, 37, and 42 °C) using a 24-well plate growth model [42] and maintaining temperature and light conditions in a Fytoscope FS130 (Photon Systems Instruments, Drasov, Czech Republic). Saline media (100 mL) were prepared by dissolving NaCl (0.5–3 g) in BG-11 media and using sterile-filtering before use. For these experiments, 1 mL cultures were grown in quadruplicate wells of a sterile 24-well plate (ThermoFisher Scientific, Waltham, MA, USA) under cool white LEDs (20 mmol/m^2^ s) and aerated by orbital rotation [42]. Growth was monitored for five days by direct chlorophyll fluorescence measurement (ex.450/em.680) in an Infinite 200 Pro plate reader (Tecan USA, Morrisville, NC, USA) and correlation to manual counts [28].

### 2.9. Soil Treatment and Biodiversity Analysis

Two 6-week pot culture experiments were conducted in an outdoor greenhouse to examine the microbial impact of amending soil with an *XB* slurry [43] and to evaluate *XB* soil persistence in outdoor conditions. In these studies, 5-gallon pots containing 16 L of potting soil (compost/vermiculite/sand, 4:1:1) over 2 L of perlite for drainage were maintained and sampled weekly during September and October (Fall experiment) and from late January through early March (Spring experiment). The average temperatures during the two experiments were 25–31 °C for the Fall experiment and 16–22 °C for the Spring experiment. The amendment plan was tested in triplicate pots, adding (a) 150 mL sterile deionized water, or (b) 150 mL *XB* culture with a cell density of 1 × 10^7^ cells/mL, or (c) 150 mL filter sterilized *XB* culture supernatant (spent media). Soil samples (10 g) were obtained weekly from each pot in sterile 15 mL tubes and stored at −20 °C, then partitioned for monitoring of pH and total dissolved solids (TDS). The starting soil mixture and endpoint (week 6) soil sample were both sampled separately (10 g) for immediate use in algal persistence checks (5 g) and microbial diversity analysis (1 g) and for storage at −20 °C.

Soil persistence was examined with *Desmodesmus XB* qualitatively by suspending 5 g pot soil in 50 mL sterile water and inoculating the resulting suspension into BG11 liquid media in 6-well plates and onto 1% agar made with BG11 medium. Microscopic verification of the regrown culture as *XB* was taken as qualitative evidence of *XB* cell survival in soil.

Functional microbial diversity in soil samples was examined using the previously reported [44,45,46] Community Level Profiling approach of the EcoPlate assay (Biolog Inc., Hayward, CA, USA). In this assay, microbial communities in the soil sample were exposed to 31 different carbon sources in triplicate and in the presence of the metabolic dye, tetrazolium blue, in a 96-well plate format. The reduction of the tetrazolium to a purple formazan was monitored at 590 nm using an Infinite 200 Pro plate reader (Tecan USA, Morrisville, NC, USA) as an indication of carbon source usage by the microbial community in the sample [45,47]. EcoPlate analyses were performed for each pot in both experiments using a modification of the manufacturer’s guidelines. Briefly, 1 g of soil from each pot was suspended in 9 mL sterile phosphate-buffered saline (PBS) and twice diluted (1:10) in sterile PBS to produce the test suspension for an individual pot. A single test suspension (0.15 mL) was applied to each well of one EcoPlate and incubated at room temperature for 5–7 days with daily monitoring of tetrazolium reduction. In accordance with previous reports [46,47], absorbance data obtained up to and including 120 h of incubation provided information about both the rate of carbon usage and the type of carbon sources used, so the incubation timepoint closest to 120 h was used for the assessment of microbial functional diversity and statistical analyses. Soil parameters of diversity including the average well color development (AWCD) score and the Shannon diversity index (H′) were calculated as described previously [45,47] using Equations (6) and (7):

(6) $$AWCD=\sum \frac{C_{i}}{93}$$

(7) $$H^{\prime }=-\sum p_{i}(lnp_{i})$$

where c_i_ is the blank-corrected OD readings for all the substrates on the plate at each timepoint, and p_i_ is c_i_ divided by the sum of all the c_i_ values for a given plate.

### 2.10. Statistical Analysis

Pearson correlation analysis was performed on standard curves for the compositional analyses of biomass and for algae cell density correlation analyses described in Section 2.2 using GraphPad Prism, version 8.00 for Windows (GraphPad Software, San Diego, CA, USA). In all cases, the number of replicates for a particular analysis is described in the Section 2, describing the analysis, and *p*-values < 0.05 were considered statistically significant.

## 3. Results

### 3.1. Isolation and Identification of a Desmodesmus Species from the Blue Hole

**Figure 3:**
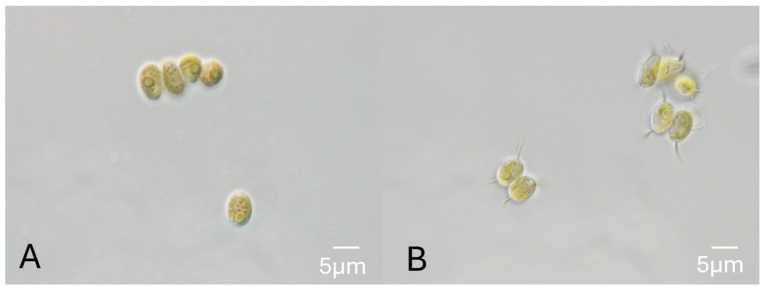
Morphology of axenic *Desmodesmus* sp. from the Blue Hole. Differential interference contrast micrographs of *AxB* cells from (**A**) agar culture and (**B**) liquid culture.

**Figure 4:**
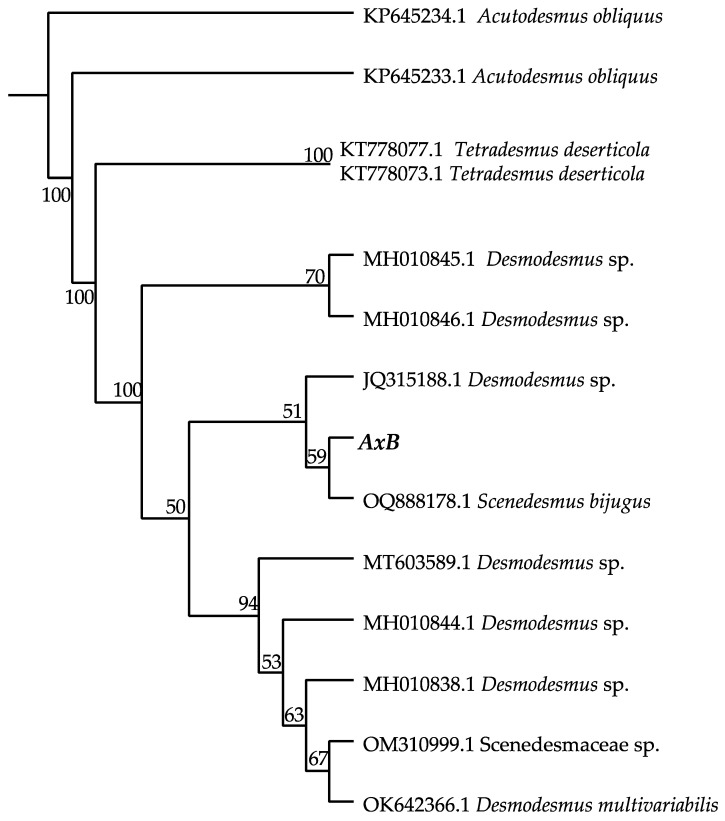
Neighbor joining tree (10,000 bootstrap trials) showing the placement of strain *AxB* in the Desmodesmus clade. The Genbank accession numbers and taxa designations are included for completeness, and bootstrap percentages are shown at the branch nodes.

A microalga was isolated by enrichment from the largest of the San Antonio Springs, the Blue Hole, in San Antonio, Texas, and established as a xenic culture, designated *XB*. Iterative single-colony selection and subculturing from the *XB* culture produced an axenic isolate, *AxB*, which was used for unambiguous DNA profiling and morphology analysis by the UTEX Culture Collection. On microscopic examination using differential interference contrast (DIC), the axenic isolate, *AxB*, presented with green, ovoid cells about 5–7 µm long with prominent pyrenoids, occurring as both unicells and coenobia of two to four cells (Figure 3). The terminal cells of *AxB* coenobia from the liquid culture displayed prominent spines, but these spines were reduced or absent in the coenobia from the *AxB* culture grown on agar medium (Figure 3). These observations indicated that *AxB* has characteristics that are consistent with the morphology of *Desmodesmus* spp. The morphology of *XB* appeared similar to *AxB* during routine light microscopy observations but with slightly more 4-cell coenobia and fewer individual cells than *AxB* (Supplementary Figure S1). DNA profiling using the ITS1 and ITS2 rDNA sequences as previously described [31] corroborated the morphology finding, indicating a high similarity (>99% identities) to strains of *Desmodesmus* spp. Phylogenetic tree construction using neighbor joining and maximum likelihood analyses (Figure 4) also indicated that the axenic isolate, *AxB*, is most closely related to strains of *Desmodesmus* sp.

### 3.2. Growth Characteristics and Biomass Composition

**Figure 5:**
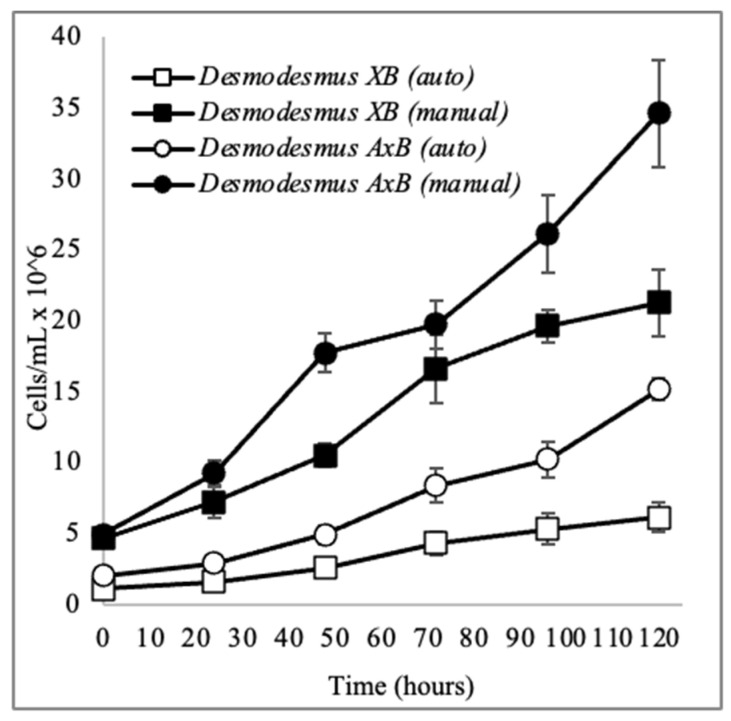
Initial growth curves for *Desmodesmus XB* and *Desmodesmus AxB* observed using both a cell counter (auto) and a Neubauer chamber (manual).

The specific growth rate and doubling times have been calculated for both the xenic and axenic cultures, *Desmodesmus XB* and *Desmodesmus AxB*, using an exponential growth period and procedures as described in Section 2.2 and Section 2.3 (Table 1). The cell density on Day 5 of the growth experiment is provided in Table 1 for reference and was determined using manual counting; however, culture densities were monitored for these experiments with both manual and automatic counting (Figure 5). Automatic quantitation of *XB* and *AxB* was observed to count coenobia as individual cells rather than as groups of cells, so both manual and automatic counting methods were used for comparison in growth experiments. Both fresh and dry weights of biomass produced by each culture were determined by harvesting growth cultures on the 5th day (Table 1).

Biomass compositions of carbohydrate, protein, and lipid were determined on stock cultures of *Desmodesmus XB* and *Desmodesmus AxB* as described in Section 2.4 (Table 1), and estimates of chlorophyll and carotenoid content were calculated from spectroscopic analysis using either the equations described by Chen and Vaidyanathan [32] or those of Wellburn (given in brackets) [35].

### 3.3. Pigment Profiles

**Figure 6:**
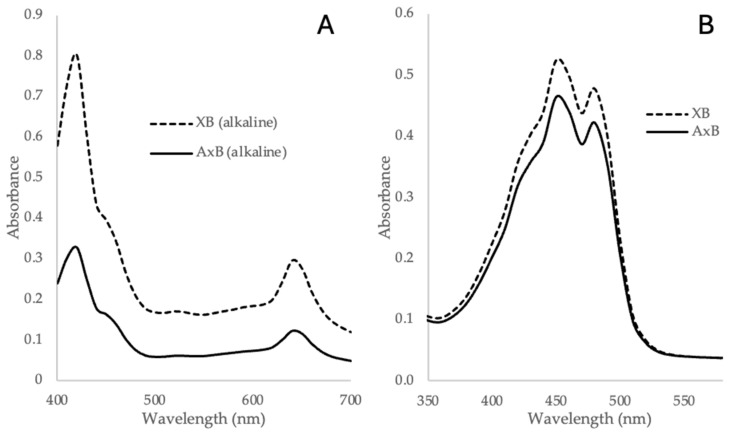
Absorbance spectra of extracts of *Desmodesmus XB* and *Desmodesmus AxB*. (**A**) The aqueous phase extracts described in Figure 2 showing chlorophyll absorbance maxima; (**B**) the organic phase extracts described in Figure 2 showing carotenoid absorbance maxima.

Chlorophylls and carotenoids were separated using the alkaline saponification method described in Section 2.5, and their estimated content in *XB* and *AxB* biomass is provided in Table 1 as calculated using the assumptions and extinction coefficients presented in Chen and Vaidyanathan [32]. Representative spectra for the chlorophyll fraction (aqueous phase after R2 extraction, Figure 2) and the carotenoid fraction (organic phase after R2 extraction, Figure 2) of *Desmodesmus XB* and *Desmodesmus AxB* are shown in Figure 6. As described by the authors of the method [32], the alkaline conditions of the method converted the chlorophylls present to magnesium-rhodochlorins (Mg-RChls), which was necessary to make a more polar chlorophyll that partitioned to the aqueous phase but which also altered the absorption spectra of the chlorophyll requiring the determination of new extinction coefficients for chlorophylls a and b and the use of new equations to calculate chlorophyll content. For application of this method to a new alga with an unknown pigment profile, it was necessary to confirm the chlorophyll and carotenoid constituencies using one or more standard pigment analysis methods for confirmation. To verify the chlorophyll content determined using the alkaline extraction method, a standard extraction and assay method lacking alkaline saponification [35] was used as described in Section 2.5, and the chlorophyll content derived from this assay is presented in Table 1 in brackets for *XB* and *AxB.* A comparison of the absorbance spectra for the two types of chlorophyll samples (alkaline method samples and standard method samples) derived from *Desmodesmus XB* and *Desmodesmus AxB* is provided in Supplementary Figure S2.

**Figure 7:**
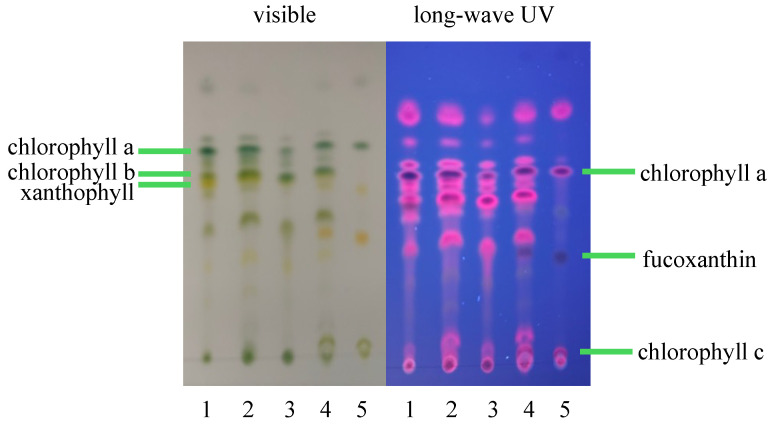
TLC comparison of extracts from *Desmodesmus AxB* with that of the haptophyte, *T. lutea*, which has chlorophylls a and c, and the more closely related chlorophyte, *S. dimorphus*. TLC, lanes: (1) *S. dimorphus*, (2) *S. dimorphus*/*AxB* co-spot, (3) *AxB*, (4) *T. lutea/AxB* co-spot, (5) *T. lutea*.

To further examine the chlorophyll and carotenoid profiles of *Desmodesmus XB* and *Desmodesmus AxB*, the reference method described in Section 2.5 was modified to extract 10 mL of stock culture rather than 2 mL. This modification provided a more concentrated extract appropriate for thin-layer chromatography (TLC) analysis [36,37,38] methods. Normal phase plates were used with a nonpolar solvent mixture of hexanes/acetone (7:3), and the results are depicted in Figure 7.

Reference algae, *T. lutea*, *S. dimorphus*, and *P. mierengensis,* for which chlorophyll profiles have been previously reported [39,40,41], were extracted using the conditions and procedures described for *XB* and *AxB* and were included in TLC analysis for comparison. Absorbance spectra were also obtained on diluted TLC samples from *XB* and *AxB* and two reference algae, *T. lutea* and *S. dimorphus*, and the comparison of the spectra is provided as Supplementary Figure S3.

### 3.4. Salt and Temperature Tolerance

**Figure 8:**
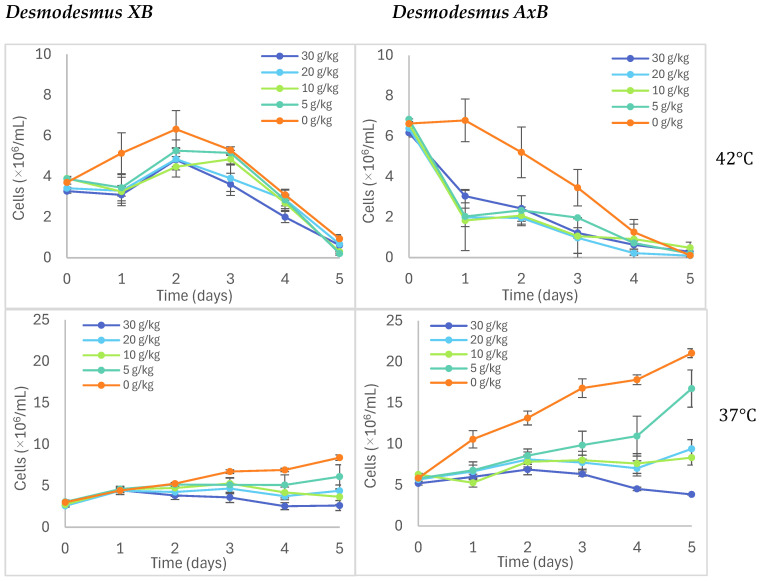

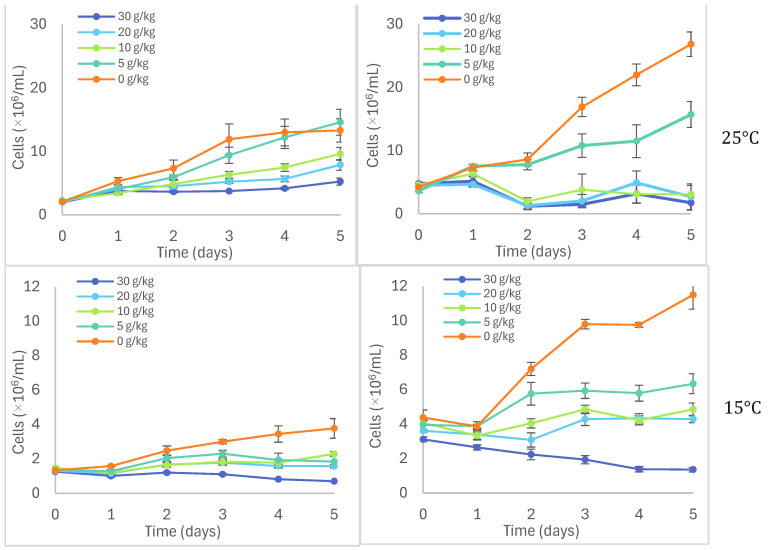
Saline growth responses in *Desmodesmus XB* and *Desmodesmus AxB* at a range of seasonal Texas temperatures.

The growth of *Desmodesmus XB* and *Desmodesmus AxB* was examined at four different temperatures and four different saline levels using a 24-well plate growth model [42] as described in Section 2.8 and summarized in Figure 8. Conditions were tested in quadruplicate wells for each isolate in BG-11 media with added NaCl at concentrations of 5, 10, 20, and 30 g/kg to test a range of salinities from weakly saline freshwater to almost marine levels. All saline and control (0 g/kg) media conditions were evaluated in separate experiments at temperatures specified in Figure 8 and Supplementary Figure S4. Cell densities were determined in these experiments by directly reading chlorophyll (Chl) fluorescence and correlating relative fluorescence to a manually counted cell number, as described in Section 2.2. The comparison of *XB* and *AxB* growth at different temperatures is depicted for the control (0 g/kg or no salt added) in Supplementary Figure S4 and as part of the saline media experiment results in Figure 8. Both isolates show an apparent growth preference for 25 °C with slower initial growth in 37 °C, slower growth overall at 15 °C, and significant die-off at 42 °C. As shown in Figure 8, the xenic isolate took longer to show loss of fluorescence at 42 °C than the axenic strain. In terms of their salt response, *AxB* exhibited a slightly higher sensitivity than *XB* to saline concentrations above 5 g/kg at all temperatures tested.

### 3.5. Soil Studies

The persistence of *Desmodesmus XB* and its short-term impact on soil microbial activity and functional diversity were examined in two triplicate pot culture experiments as described in Section 2.7. A live culture of *Desmodesmus XB* was amended to the top 6 cm of soil as a slurry in deionized water and compared with identically amended deionized water or sterile-filtered media from *XB* culture (no cells present) slurried in the same ratio as the culture in deionized water. This design was adopted to allow for the effects of algal media components to be considered separately from the cells in the media and gave a slightly more rigorous test of *XB* survival in soil. The differences in the two trials were primarily in average temperature (10–14 °C difference) and lighting (seasonal differences in intensity and duration).

#### 3.5.1. Soil Persistence

**Figure 9:**
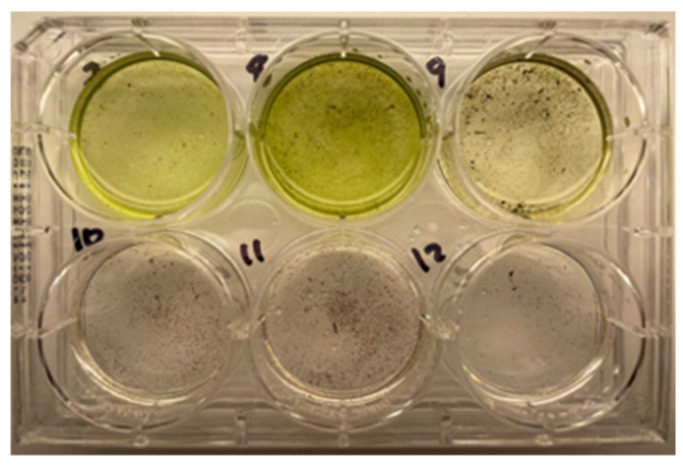
The short-term persistence of *Desmodesmus XB* in soil. The numbers on the plate represent pot numbers. The top row of wells contained soil dilution from pots amended with *XB* cells. The bottom row of wells contained soil dilution from pots amended with sterile filtered media from *XB* culture. The green color indicates regrowth of algae from the soil sample.

The ability of *Desmodesmus XB* to survive for 6 weeks in a soil community was examined by applying a serial dilution of 6-week soil either solid BG11 medium (1% agar) or liquid BG11 medium and incubating the inoculated medium in light for 2–3 days to observe regrowth of *Desmodesmus XB*. Figure 9 shows the typical regrowth of *Desmodesmus XB* from fresh 6-week soil samples and the lack of regrowth in soil samples from pots without *Desmodesmus XB* cells added. Examples of regrowth on 1% agar BG11 are shown in Supplementary Figure S5.

#### 3.5.2. Soil Microbial Activity and Functional Diversity

Carbon substrate utilization, assessed quantitatively and qualitatively using Biolog EcoPlates as described in Section 2.7, showed modest differences in soil treated with *Desmodesmus XB* slurry, sterile-filtered *XB* growth media, or sterile-filtered water, as summarized in Table 2. The AWCD was highest in both seasonal experiments for soil amended with *Desmodesmus XB* cells, but soil treated with sterile-filtered *XB* growth media was also higher in overall microbial activity by the measure of the AWCD than the control soil amended with water. These results suggest that living *Desmodesmus XB* in the soil community strengthened its metabolic activity. The Shannon diversity index (H′) was calculated as described in Section 2.7 and applied to the soil study data as a measure of how functionally diverse a soil microbial community is based on the breadth carbon sources it uses [46]. The AWCD and H′ parameters were calculated and compared for both soil experiments, and as shown in Table 2, microbial activity differences among the treatment conditions were modest, and H’ were not outside experimental error for any compared conditions.

## 4. Discussion

### 4.1. Morphological Identification and Phylogenetic Analysis

The initial microalgal isolate obtained from the Blue Hole by enrichment and designated *XB* was purified to obtain an axenic culture, designated *AxB*, which was identified as a *Desmodesmus* sp. based on morphological features and phylogenetic analysis. The morphological features of both the xenic and axenic cultures of the studied strain (Figure 3) align well with *Desmodesmus* morphology, which includes prominent pyrenoids; multicell coenobia with terminal spines observed in liquid culture; and fewer spines, if any, observed on agar [48]. As part of the identification process, a phylogenetic tree was obtained using conventional methods (Figure 4), which placed the *AxB* isolate in the Chlorophyceae class, Sphaeropleales order, and Scenedesmaceae family, with closest relatives including other Desmids, a *Scenedesmus bijungus* isolate, and some potential extremophiles [18]. The xenic isolate was initially maintained as a backup to the axenic *Desmodesmus* culture. However, recent reports on potential applications of algal consortia in soil remediation and soil health maintenance [9,14] encouraged the study of *XB* alongside *AxB* in terms of growth parameters, biomass constituencies, and soil persistence.

### 4.2. Growth Characteristics

Under the conditions tested, the new *Desmodesmus XB* and *Desmodesmus AxB* exhibited doubling times of 1.53 and 1.02 days, respectively (Table 1). Faster growth rates observed for axenic microalgal cultures relative to their xenic cultures have been reported [49]. More often, however, natural isolates that are grown in laboratory conditions with their native microbial partners present in culture exhibit faster growth rates and altered culture behaviors relative to their more isolated, axenic counterparts [50,51,52,53], presumably due to organically established microalgal–bacterial cooperativity [52]. In the case of *XB* and *AxB*, several laboratory observations reflected slightly different culture behaviors in the cultures. Under routine cultivation, *XB* cultures settled more rapidly without aeration than *AxB* cultures, and harvesting by centrifugation generally resulted in fluffier, less well-formed biomass pellets from *XB* cultures than from *AxB* cultures.

Like other desmids, both *XB* and *AxB* tend to form coenobia, which complicate the use of automatic cell counters because they are variably counted as single cells rather than as groups of cells. Cell densities were therefore monitored using manual counting correlated to either automatic counting or chlorophyll fluorescence depending on the format of the experiment. For growth experiments on the one-liter scale, automatic counting is fast and convenient as a correlate (Figure 5), and for the 24-well plate growth studies on sensitivity to saline conditions and temperature (Figure 8), the direct reading of fluorescence is extremely facile and is less impacted than optical density measurements by the observed well growth anomalies in both xenic and axenic cultures of filming and clumping.

### 4.3. Salt and Temperature Tolerance

While optimal growing temperatures for most microalgal species range from 14 °C to 30 °C, microalgae that are promising for agricultural and environmental applications require robust growth at higher temperatures. High culture temperatures have been reported in outdoor raceway ponds and other industrial scale cultivations [54,55], and higher outdoor temperatures in growing locales such as Texas reflect higher soil temperatures that could impact the soil survival of applied microalgae. To determine whether *XB* and *AxB* would be more tolerant of higher temperatures as native Texas algae, growth of both isolates was observed at temperatures corresponding to the cooler temperatures of autumn and winter in Texas (15 °C); the standard laboratory temperature (25 °C); and the often-seen spring and summer temperatures in Texas, 37 °C and 42 °C, respectively. Surprisingly, both isolates were unable to withstand 42 °C (Figure 8), although a lag in decolorization was observed for *XB* that was not observed for *AxB*. At the other temperatures, *XB* and *AxB* had similar growth profiles with peak growth rates at 25 °C and slowest positive growth rates at 15 °C. Both *XB* and *AxB* demonstrated robust growth at 37 °C, which is outside the optimal growing temperature range and somewhat expected for an alga native to such a hot, dry climate. Higher temperature growth levels (42–45 °C) have been reported in other new microalgal isolates [55,56].

Tolerance of high-saline growth environments is a desirable trait in microalgae that are useful for soil and water remediation [57] since both applications involve saline levels that exceed typical freshwater saline (0.02–1 g/kg). Both *XB* and *AxB* were unable to sustain growth in saline media at or above 10 g/kg NaCl; however, both showed robust growth in 5 g/kg NaCl, which is on the lower side of the salinity range found in industrial wastewaters [57]. Slightly higher resistance to saline growth media is expected since regular dehydration and hydration periods of the seasonal San Antonio Springs system would likely encourage the development of high saline tolerance in its native microalgae [12].

### 4.4. Compositional Biomass Analysis

Numerous methods for compositional analysis of microalgal biomass are available featuring the use of dry or fresh biomass, a myriad of solvent combinations, and almost as many cell disruption methods [58]. For this work, an economical and rapid method was selected to provide robust estimates of algal biomolecules that would use fewer lab resources and streamline the flow of samples (Figure 2) and analysis methods [32]. The xenic and axenic *Desmodesmus* cultures were not expected to exhibit significant differences in carbohydrate, protein, and lipid content, and indeed both isolates were within the ranges of these components predicted for *Desmodesmus* species [27].

The classification of *Desmodesmus* would tend to predict the presence of chlorophylls a and b and a selection of carotenoids and xanthophylls [40,59] in *XB* and *AxB* biomass. Accordingly, the chlorophyll and carotenoid constituents of both *XB* and *AxB* were examined using the convenient alkaline saponification extraction and combination assay method of Chen and Vaidyanathan [32] (Figure 2) and were also examined using a more traditional method of extraction in 80% acetone [35]. The two methods provided excellent agreement for chlorophyll content but less so for carotenoid content (Table 1). Absorption spectra for the standard or reference method extracts and the alkaline method extracts of *XB* and *AxB* (Figure 6) demonstrated the predicted shifts in chlorophyll a and b maxima [32] and the carotenoid peaks described previously [32]. Taken together, the data suggest that the chlorophyll and carotenoid estimates presented here provide an accurate pigment profile of this new *Desmodesmus* species. For visualization and qualitative assessment of the chlorophylls and carotenoids present in *Desmodesmus AxB*, a more concentrated extract was prepared in 80% acetone and subjected to TLC analysis (Figure 7). Extracts were similarly prepared for TLC analysis from a related freshwater desmid, *Scenedesmus dimorphus*, and the marine haptophyte, *Tisochrysis lutea*, to provide comparative TLC profiles that include chlorophylls a and b (*S. dimorphus*) and chlorophyll c_1_ (*T. lutea*). The close agreement in chlorophyll profiles of *AxB* and *S. dimorphus* provides further support for the phylogenetic and morphological assignment of *AxB* as *Desmodesmus*.

### 4.5. Soil Persistence and Impact on Soil Microbiome

Although the *XB* isolate is a native Texas alga and has demonstrated robust growth at 37 °C in liquid culture, some information about the survival of the new *Desmodesmus* sp. in agricultural soil and higher temperature conditions is necessary to establish soil studies on a field scale in Texas. The preliminary soil experiments presented here have confirmed that *Desmodesmus XB* does persist in pot culture soil (Figure 9). However, the impacts on the soil microbiome observed in the community-level profiling of *XB*-amended soil were modest compared with unamended soil, both in terms of increased microbial activity and enriched soil diversity (Table 2). Given the longer-term nature of the profiling experiments reported previously [46,47], it is likely that the limited increases in microbial activity and diversity associated with *XB* amendment reflect the short time period of the experiment and could argue for a longer-term study (20–52 weeks) that includes *AxB* as well as *XB* and incorporates multiple inoculation levels of microalgal slurry. Future soil studies may also examine the ability of the new *Desmodesmus* isolates to impact microbial activity and diversity in degraded soils and to interact in vitro with specific plant beneficial microbes that support plant and soil health.

## 5. Conclusions

A novel microalgal isolate from the San Antonio Springs has been identified as a *Desmodesmus* species using morphological and phylogenetic analyses. The new *Desmodesmus* has been characterized in both xenic culture, designated *XB*, and axenic culture, *AxB*, with respect to growth in standard media, growth in saline and thermal stress, and biomass composition. The *AxB* growth rate in BG-11 media exceeds that of *XB* by a significant margin, and both *XB* and *AxB* tolerate 5 g/kg saline media and grow robustly at 37 °C in liquid culture. Both the xenic and axenic cultures demonstrate morphology and biomass constituencies of carbohydrate, protein, and lipid that are consistent with other members of the *Desmodesmus* genus. Like other desmids, the *XB* and *AxB* exhibit chlorophylls a and b and display similar carotenoid profiles to a reference desmid, *S. dimorphus*, as confirmed spectrally and chromatographically. *Desmodesmus XB* persisted in potting soil in both low- and high-temperature pot culture studies, and modest impacts on soil microbial activity and functional diversity were observed in *XB* amended soil. This research highlights the potential advantages in bioprospecting for native microalgae for potential use in sustainable and efficient agriculture.

## Figures and Tables

**Table 1:** Growth characteristics and biomass composition of *Desmodesmus XB* and *Desmodesmus AxB*.

Growth or Biomass Parameter	*Desmodesmus XB*	*Desmodesmus AxB*
Specific growth rate m (d^−1^)	0.46 ± 0.042	0.77 ± 0.03
Doubling time (d)	1.53 + 0.069	1.02 ± 0.15
Cell number (×10^6^ cells/mL)	17.6 ± 4.2	29.6 ± 6.1
Dry weight (DW, mg/L)	441.3 ± 72.0	488.7 ± 81.9
Carbohydrate (% DW)	21 ± 6.3	13 ± 4.1
Protein (% DW)	50 ± 0.9	49 ± 0.4
Lipid (% DW)	32 ± 3.4	28 ± 0.3
Chlorophylls (mg/L) *	11.7 [11.4]	13.8 [14.0]
Carotenoids (mg/L) *	2.6 [1.1]	2.2 [0.9]

* Standard pigment assay method [35] result in brackets.

**Table 2:** Community-level profiling of short-term soil studies.

Parameter	Soil Treatment	September–October (Fall)	January–March (Spring)
AWCD (120 h)	Water	1.66 ± 0.03	1.46 ± 0.09
	Spent medium	2.09 ± 0.12	2.32 ± 0.16
	*XB* cells	3.96 ± 0.24	3.26 ± 0.17
Shannon diversity index (120 h)	Water	2.90 ± 0.11	2.92 ± 0.06
	Spent medium	3.00 ± 0.07	2.91 ± 0.07
	*XB* cells	3.06 ± 0.05	2.97 ± 0.07

## Data Availability

Data supporting this work are available upon request from the corresponding author.

## Supplementary Materials

The following are available online at https://www.mdpi.com/article/10.3390/life14091053/s1. Figure S1. The appearance of *Desmodesmus XB* and *Desmodesmus AxB* appear to be similar at the magnification used for cell counting, but XB appears to have slightly more coenobia that are groups of four than AxB does. Figure S2. Absorbance spectra of pigments extracted from *Desmodesmus XB* and *Desmodesmus AxB* using the alkaline method [CHEN 2013] or the reference method with 80% acetone [CHEN 2013]. Figure S3. Absorbance spectra of pigments extracted from *Desmodesmus XB* and *Desmodesmus AxB* and from reference algae *T. lutea* (contains chlorophyll a and chlorophyll c_1_) and *S. dimorphus* (contains chlorophyll a and chlorophyll b). Figure S4. Growth responses to temperature of *Desmodesmus XB* and *Desmodesmus AxB.* Figure S5. Examples of soil regrowth on 1% agar made with BG11.
