# Supplementary material for: Growth Characteristics of a Desmodesmus Species from the San Antonio Springs and Its Short-Term Impact on Soil Microbial Dynamics
Source: Life (Basel). 2024 Aug 23;14(9):1053. doi: 10.3390/life14091053 (PMC11433310; doi:10.3390/life14091053)
Supplement: Supplementary file 1 [file life-14-01053-s001.zip › life-3125568-supplementary.pdf]

Growth Characteristics of a *Desmodesmus* Species from the San Antonio Springs and Its Short-Term Impact on Soil Microbial Dynamics

SUPPLEMENTARY MATERIALS

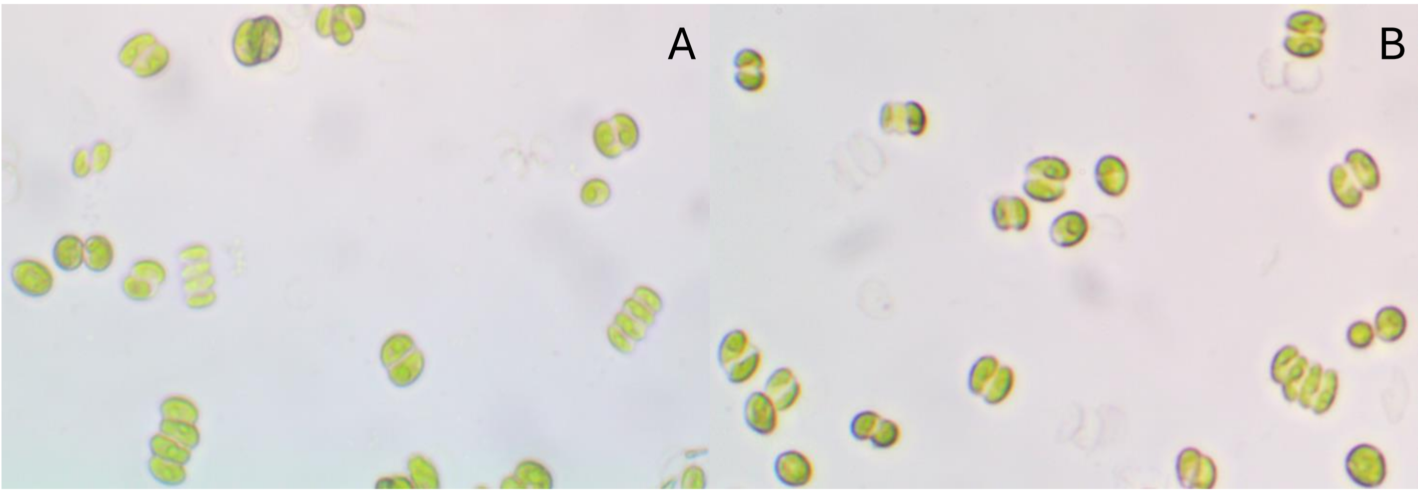

Figure S1. The appearance of ***Desmodesmus XB*** and ***Desmodesmus AxB*** appear to be similar at the magnification used for cell counting, but XB appears to have slightly more coenobia that are groups of four than AxB does.

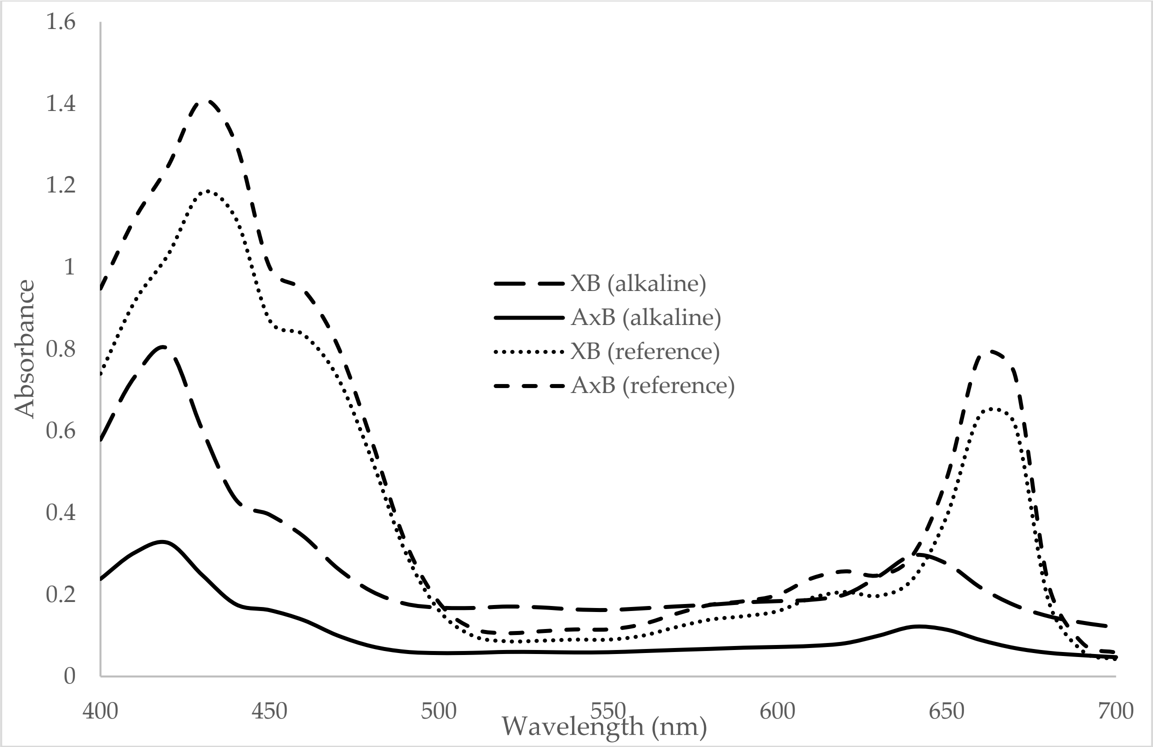

Figure S2. Absorbance spectra of pigments extracted from ***Desmodesmus XB*** and ***Desmodesmus AxB*** using the alkaline method [CHEN 2013] or the reference method with 80% acetone [CHEN 2013].

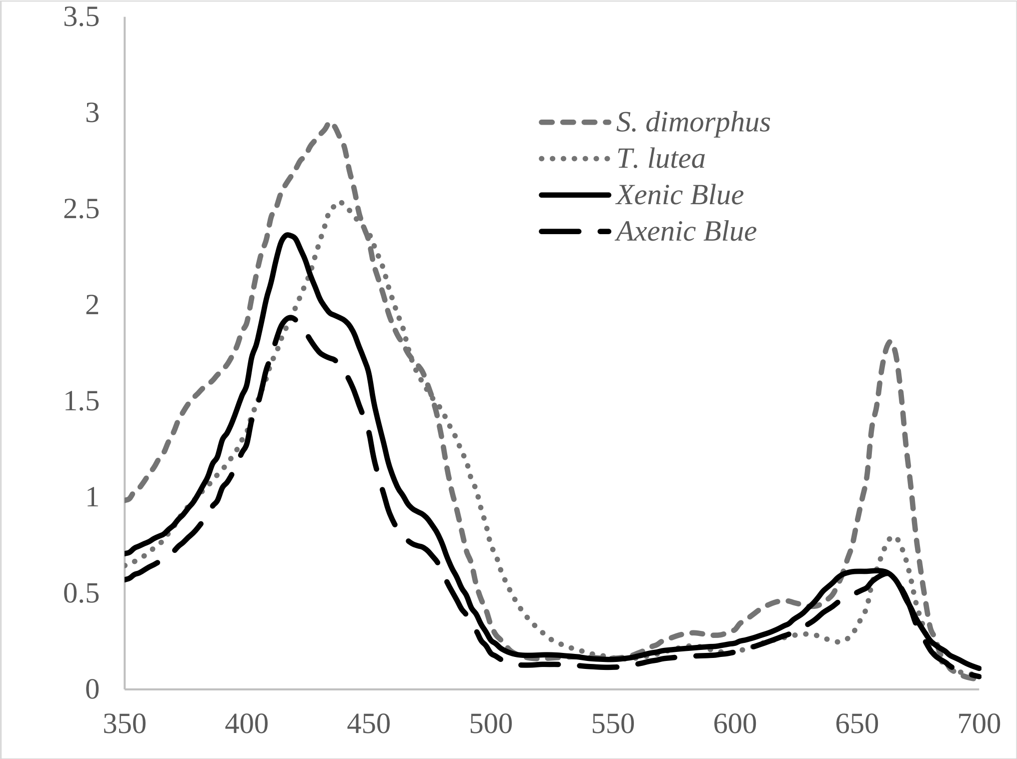

Figure S3. Absorbance spectra of pigments extracted from ***Desmodesmus XB*** and ***Desmodesmus AxB*** and from reference algae *T. lutea* (contains chlorophyll a and chlorophyll c_1) and *S. dimorphus* (contains chlorophyll a and chlorophyll b).

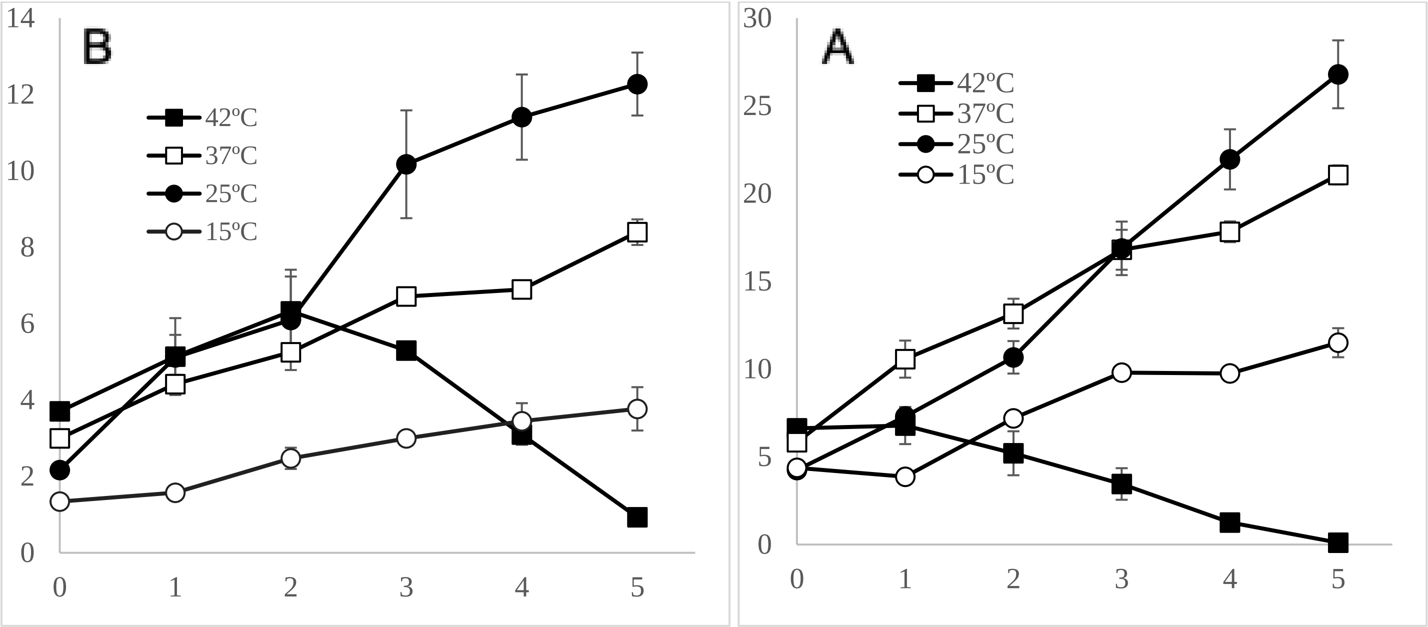

Figure S4. Growth responses to temperature of ***Desmodesmus XB*** and ***Desmodesmus AxB.***

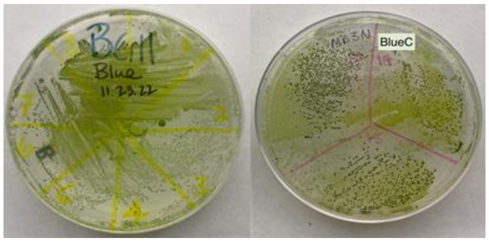

Figure S5. Examples of soil regrowth on 1% agar made with BG11.
